# Functional Chewing Gum Enriched with Spent Coffee Grounds Extract: Chemical Characteristics and Antimicrobial Potential Against Oral Bacteria

**DOI:** 10.3390/foods15040640

**Published:** 2026-02-10

**Authors:** Hanem M. M. Mansour, Elsayed E. Hafez, Mahdy N. Elnaggar, Hager S. A. Alsonpaty, Salim A. Ali, Asteer V. Abd-Elnoor, Amira A. Abdellatef, Omayma E. Shaltout, Marwa G. Allam, Eman M. Abdo

**Affiliations:** 1 Food Technology Department, Arid Lands Cultivation Research Institute (ALCRI), City of Scientific Research and Technological Applications (SRTA-City), New Borg El Arab P.O. Box 21934, Alexandria, Egypt; hmahmoud@srtacity.sci.eg (H.M.M.M.); or mahdy@scut.edu.cn (M.N.E.); 2 Plant Protection and Biomolecular Diagnosis Department, Arid Lands Cultivation Research Institute, City of Scientific Research and Technological Applications (SRTA-City), New Borg El Arab P.O. Box 21934, Alexandria, Egypt; ehafez@srtacity.sci.eg; 3 School of Food Science and Engineering, South China University of Technology, Guangzhou 510640, China; 4 Microbiology Department, Faculty of Science, Tanta University, Tanta P.O. Box 31511,Tanta, Egypt; hagersaad2992912@gmail.com; 5 Date Palm Research Center of Excellence, King Faisal University, P.O. Box 400, Al-Ahsa 31982, Saudi Arabia; 6 Home Economics Department, Nutrition & Food Science, Faculty of Specific Education, Alexandria University, Alexandria P.O. Box 21648, Alexandria, Egypt; ester.vector@alexu.edu.eg; 7 Department of Protein Technology, Institute of Genetic Engineering and Biotechnology, City of Scientific Research & Technological Applications, New Borg El Arab P.O. Box 21934, Alexandria, Egypt; 8 Food Science Department, Faculty of Agriculture (Saba Basha), Alexandria University, Alexandria P.O. Box 21531, Alexandria, Egypt; prof.dr.omimaelsaidshaltout@alexu.edu.eg (O.E.S.); marwa.g@alexu.edu.eg (M.G.A.)

**Keywords:** bioactive compounds, oral bacteria, *Streptococcus mutans*, frankincense, biodegradable gum, functional food

## Abstract

Spent coffee grounds (SCGs), as a by-product, represent a sustainable source of bioactive components. Herein, the ethanol extract of SCGs was examined for its chemical characteristics and antimicrobial effect. The extract was incorporated into novel frankincense-based gums at concentrations of 0% (T0), 15% (T15), 20% (T20), and 25% (T25). The physicochemical properties and the antibacterial activity of the SCG-enriched gums against oral pathogens were assessed. SCG extract showed a total phenolic content of 999.38 ± 2.63 μg/g and demonstrated antioxidant activity with a 50% inhibitory concentration (IC_50_) of 107.28 ± 1.90 μL/mL. T25 showed the highest phenolic content (256.66 ± 2.93 μg/g) and enhanced scavenging activity IC_50_ = 211.05 ± 0.65 (DPPH) and 128.52 ± 4.05 μL/mL (ABTS). T25 demonstrated superior antimicrobial effects against *Streptococcus mutans* and *Enterococcus faecalis* at 400 µL/mL, with inhibition zones of 33.33 ± 2.89 and 20.33 ± 0.58 mm compared to T0. Both T25 and T0 inhibited *Lactobacillus acidophilus* similarly. Overall, incorporating SCG extract into natural frankincense-based gum presents a promising biodegradable functional gum with potential oral health benefits.

## 1. Introduction

Functional foods are those that provide basic nutrition and confer additional health benefits due to their biologically active components. The increasing interest in functional foods has encouraged researchers to incorporate diverse bioactive ingredients to promote human health. Plant and food industry by-products have been widely utilized in developing functional foods due to their richness in bioactive components [1]. Moreover, using by-products aligns with consumer preferences for natural additives and supports sustainability by conserving natural resources [2], and mitigating the environmental impact of food waste [3].

Spent coffee grounds (SCGs), a by-product of coffee brewing, are generated at approximately 0.91 g per gram of ground coffee used [4]. SCGs are often discarded in landfills or utilized as fertilizers or feedstocks [3]. However, SCGs are a valuable source of fiber, proteins, lipids, essential minerals, and phytochemicals, such as caffeine, chlorogenic acid, and melanoidins [4]. Therefore, upcycling SCGs represents an eco-sustainable strategy to reduce the carbon fingerprint [1] and global warming potential [3], while developing functional products.

SCGs have been incorporated into the production of various products, including biscuits, muffins, bread, cake, and cookies, improving their nutritional value. However, sensory responses to SCG addition varied depending on the product; cookies were the most receptive to SCGs [1]. Beyond nutritional improvement, SCGs showed notable health-promoting effects. SCG extract has been reported to attenuate H_2_O_2_-induced oxidative stress in *Centropomus viridis* brain cells [5]. Moreover, biscuits enriched with SCGs have been shown to mitigate obesity by increasing satiety [6] and modulating gut microbiota composition [7]. Therefore, valorization of SCGs in suitable applications not only adds value to products but also promotes human health and contributes to the sustainability goals.

Chewing gum is a widely consumed product made using a rubbery base. Conventional chewing gums mainly rely on using petroleum-derived gum bases, which are synthetic, non-biodegradable, and harmful to the environment [8]. Consequently, the use of natural, biodegradable gum base has recently gained attention because of its lower environmental impact [8]. Among them, frankincense (*Boswellia serrata*) resin is commonly used in the Middle East as gum with potent anti-inflammatory and antibacterial effects [9]. Using frankincense as a gum base thus offers a promising approach to developing biodegradable and eco-friendly gum, with health benefits.

Chewing gum is not swallowed, making it an effective delivery system for therapeutic ingredients that are gradually released from the gum matrix during mastication [10]. Incorporating SCGs in chewing gum could overcome the limitations of using SCGs in traditional food applications [1]. Besides, due to the high antioxidant and antimicrobial properties of SCGs [11,12], SCG-enriched gum may offer an innovative approach to inhibit harmful oral bacteria, such as *Streptococcus mutans*, *Lactobacillus acidophilus*, and *Enterococcus faecalis*, which are associated with dental caries [13], obesity [14], and Alzheimer’s [15].

Therefore, the current study aims to characterize the SCG ethanol extract by evaluating its phenolic content, antioxidant activity, and antimicrobial effect. Furthermore, the research explores the potential application of SCGs in producing a novel chewing gum, with possible benefits for oral health. The SCG-enriched gum was subsequently assessed for its inhibitory effects against harmful oral bacteria, including *Streptococcus mutans*, *Lactobacillus acidophilus*, and *Enterococcus faecalis*.

## 2. Materials and Methods

### 2.1. Materials

Spent coffee grounds (SCGs), from Arabica coffee (*Coffea arabica*), were collected from a local cafeteria in Alexandria, Egypt, during a 2-h period of the brewing process. The SCGs were dried at 105 °C to a constant weight before extraction. Frankincense was purchased from a local market in Alexandria, Egypt. All chemicals and reagents were obtained from Merck (Darmstadt, Germany) and Aljomhoria (Alexandria, Egypt). The microbial strains were obtained from the Microbiological Resources Center (MERCIN), Faculty of Agriculture, Ain Shams University, Cairo, Egypt, and the Genetic Engineering and Biotechnology Research Institute (GEBRI), Scientific Research and Technological Applications (SRTA-City), Borg Al-Arab, Alexandria, Egypt.

### 2.2. Extraction of Spent Coffee Grounds (SCGs) Extract

SCG extract was prepared following the method of Meerasri & Sothornvit [16]. Dried SCGs were mixed with ethanol (1:50 *w*/*v*) and shaken at 180 rpm for 16 h. After that, the mixture was filtered and frozen at −28 °C for 48 h to separate the impurities. Subsequently, the pure extract was separated by centrifuging the mixture at 10,000 rpm for 1 min at 4 °C. The extract was then stored at −20 °C until further analysis and use. The extraction yield was calculated according to Equation (1).

(1) $$\mathrm{Yield}\;\%=\frac{\mathrm{Weight}\;\mathrm{of}\;\mathrm{SCG}\;\mathrm{extract}}{\mathrm{Weight}\;\mathrm{of}\;\mathrm{SCG}\;\mathrm{powder}}\times \;100$$

### 2.3. Total Phenolics of SCG Extract

Briefly, 200 µL of the extract was mixed with 1 mL of Folin–Ciocalteu reagent (0.2 N) and 800 µL of sodium carbonate (7.5%). The mixture was then incubated for 2 h in the dark before the absorbance was measured at 760 nm using a spectrophotometer (Jenway 6405UV/VIS, Stone, Staffordshire, UK). Gallic acid was used as a standard; the total phenolics were expressed as µg gallic acid/g of extract [16].

### 2.4. Total Flavonoid Content of SCG Extract

The extract (1 mL), d.H_2_O (4 mL), and sodium nitrite (300 µL; 5%) were incubated for 5 min before 300 µL of aluminum chloride (10%) was added. After 6 min, 2 mL of NaOH (1 moL/mL) was added, and the volume was adjusted to 10 mL with d.H_2_O. The absorbance was then measured at 510 nm; the flavonoids were expressed as µg/g using catechin as a standard [12].

### 2.5. Fourier Transform Infrared Spectrophotometer (FT-IR) of SCG Extract

The spectrum of the SCG extract was determined using a Fourier transform infrared spectrophotometer (Shimadzu FTIR-8400 S, Kyoto, Japan) equipped with an ATR-8000A accessory. Initially, 5 mg of the dried SCG extract was ground in a mortar with 100 mg of spectroscopic-grade potassium bromide (KBr). The mixture was then pressed using a pellet presser to obtain a thin pellet, which was subsequently scanned under the IR beam at a wavenumber range of 4000–400 cm^−1^ [16].

### 2.6. DPPH Scavenging Activity of SCG Extract

Different concentrations of the extract were prepared in ethanol (0–300 µL/mL). Each dilution (0.5 mL) was mixed with 0.5 mL of 0.3 mM DPPH (2,2, diphenyl-1-picrylhydrazyl) in ethanol and incubated for 20 min at room temperature (25 °C ± 3). The absorbance was then measured at 517 nm, and the inhibition ratio was calculated (Equation (2)). IC_50_, the concentration that inhibited 50% of the radicals, was determined using linear regression analysis and expressed in µL/mL [17].

(2) $$\%\;Inhibition=\frac{absorbance\;of\;control-absorbance\;of\;sample}{absorbance\;of\;control}\;\times \;100$$

### 2.7. Antimicrobial Effect of SCG Extract

The antimicrobial effect of SCG extract was assessed using the agar well diffusion method against two Gram-positive strains (*Clostridium perfringens* EMCC 1574 and *Streptococcus mutans* ATCC 25175), two Gram-negative strains (*Escherichia coli* BA 12296 and *Pseudomonas aeruginosa*), and a fungal strain (*Candida albicans* EMCC 105). The strains were initially incubated overnight in nutrient broth at 37 °C. Next, 1 mL of each strain (10^8^ CFU/mL) was mixed with molten nutrient agar in sterile Petri dishes. The bacterial density was adjusted to approximately 10^8^ CFU/mL by measuring the optical density at 600 nm (OD_600_). After the media solidified, four wells (6 mm) were created in each plate and pipetted with 100 μL of the extract at concentrations of 0, 25, 50, and 100 μL/mL. The applied volume (100 μL) was selected to ensure adequate diffusion of the extract within the agar without overflow from the wells. The plates were kept at 4 °C for 30 min to ensure the diffusion of the extract. Subsequently, the plates were incubated at 37 °C for 18 h, and the inhibition zones were then measured and expressed in mm [18]. Ethanol (used as the extraction solvent) was tested as a negative control under the same conditions and shoed no inhibition zone (0 mm) against the tested strains.

### 2.8. Cytotoxicity of the SCG Extract

Cytotoxicity of the SCG extract was evaluated using WI-38 human lung fibroblast cells (American Type Culture Collection, Manassas, VA, USA). The cells were cultured on Eagle’s Minimum Essential Medium (EMEM) supplemented with 10% fetal bovine serum (FBS) and 1% penicillin-streptomycin in a humidified incubator with 5% CO_2_ at 37 °C. After that, the harvested cells were seeded at 8 × 10^3^/well in a 96-well plate containing 100 µL medium and incubated for 24 h. Subsequently, the medium was removed, and the cells were treated with serial dilutions of the SCG extract in DMSO (0–1000 µg/mL) for 24 h. Cell viability was determined using the MTT assay (3-(4,5-dimethylthiazol-2-yl)-2,5-diphenyl tetrazolium bromide): MTT (20 µL, 5 mg/mL) was added to the wells and incubated at 37 °C for 4 h. The formazan crystals were then dissolved in 150 µL DMSO, and the optical density (OD) was measured at 560 nm on a microtiter plate reader spectrophotometer (SpectrostarNano, BMG Labtech). The OD was directly related to the number of cells, and the cytotoxic concentration (CC_50_) was then calculated using the GraphPad Prism version 8 (San Diego, CA, USA) [19].

### 2.9. Preparation of Chewing Gum Using SCG Extract

Before preparing the chewing gums, the ethanol was completely evaporated from the extract using a rotary evaporator to ensure that the final products were free of ethanol and safe for consumption. The chewing gum was then prepared using different concentrations of SCG extract: 0% (T0), 15% (T15), 20% (T20), and 25% (T25), following Bölük et al. [20] with modifications. Frankincense resin was chosen as a natural, biodegradable gum base, with anti-inflammatory and antibacterial effects [9]. The gum base was melted at 70 °C until it was soft and then homogenized with sorbitol and saturated sorbitol solution (Table 1). For SCG-enriched gums, starch was included as a filler to stabilize the gum texture at high concentrations of the extract [8]. The mixture was then left to cool, reaching 40 °C. Afterwards, different concentrations of SCG extract (15, 20, and 25 g) were mixed with the lecithin and added to the mixture to prepare T15, T20, and T25 formulations, respectively. Lecithin was used as an emulsifier and softener to disperse the extract through the gum dough. A control gum (T0) without SCG extract was also prepared. The samples were shaped, wrapped in wax paper, and kept at room temperature in a sealed container until analysis. The SCG percentages were determined based on preliminary experiments using the SCGs at concentrations from 5–30%.

### 2.10. Sensory Evaluation of the Formulated Gums

The sensory evaluation of the formulated gum was conducted after approval from the Ethics Committee, Faculty of Medicine, Alexandria University (Institutional Review Board No. 00012098, Serial No. 0307603). The panellists were untrained volunteers (8 males and 12 females, aged 20–60 years). The chewing gum was randomly coded and introduced to the panellists. Panellists were instructed to chew each gum (1 g) for 15 min before rating its sensory attributes, including hardness, chewiness, taste, odor, color, and overall acceptability using a 9-point Hedonic scale (1 dislike extremely and 9 like extremely) [21].

### 2.11. Color Parameters of the Formulated Gums

Color measurements were performed on the surface of the chewing gum samples by a colorimeter (HunterLab, EasyMatch QC, Reston, VA, USA) based on the CIE L* (lightness/darkness), a* (redness/greenness), and b* (yellowness/blueness). Measurements were taken at multiple points on each sample, and the mean values were reported. The values of a* and b* were used to calculate chroma (C*), hue angle (h*), and color change (ΔE * ab) according to Equations (3), (4), and (5), respectively [18].

(3) $${C^{*}=\left(a^{*2}+b^{*2}\right)}^{1/2}$$

(4) $$\mathrm{Hue}=(\tan^{-1}\times \;360)/(2\;\times \;\pi )$$

(5) $$\Delta E*\mathrm{ab}=[({L^{*}}_{I}-{L^{*}}_{0}{)}^{2}+({a^{*}}_{I}-{a^{*}}_{0}{)}^{2}+({b^{*}}_{i}-{b^{*}}_{0}{)}^{2}{]}^{1/2}$$

where L*_0_, a*_0_, and b*_0_ are color values of the control, and L*_i_, a*_i_, and b*_i_ are color values of the SCG-enriched gums.

### 2.12. Extraction of Bioactive Components from Gum

Bioactive components were extracted from the formulated gums following the method of Bölük et al. [20]. Gum samples were mixed with phosphate-buffered saline (1:2 *w*/*v*) for 10 min before the mixtures were centrifuged at 5000 rpm for 10 min. Then, the mixture was mixed with ethanol (1:9; chewing gum: ethanol) and centrifuged again at 5000 rpm for 30 min. The supernatants were collected and stored at −18 °C for further analysis.

### 2.13. Phenolic Content and Antioxidant Activity of the Formulated Gums

The gum extracts were analyzed for total phenolics and DPPH scavenging activity following the methods described in Section 2.3 and Section 2.6, respectively.

For ABTS (2,2′-azino-bis (3-ethylbenzothiazoline-6-sulfonate)) scavenging activity, ABTS reagent was prepared by incubating equal volumes of ABTS (7 mmol/L) with potassium persulfate (2.4 mmol/L) in the dark for 16 h. The reagent was then diluted with d.H_2_O (1:60 *v*/*v*) to obtain an absorbance of 0.701 ± 0.01 at 734 nm. Afterwards, the reagent (4 mL) was added to each sample (10 μL), and the mixture was then incubated for 6 min. The absorbance of the samples and control was measured at 734 nm; the ABTS radical scavenging activity was calculated according to Equation (6) [17].

(6) $$\%\;Inhibition=\frac{absorbance\;of\;control-absorbance\;of\;sample}{absorbance\;of\;control}\;\times 100$$

### 2.14. Antimicrobial Effect of SCG-Enriched Gum Against Oral Bacteria

T0 and T25 extracts were tested for their antimicrobial activity against *Streptococcus mutans* ATCC 25175, *Enterococcus faecalis* ATCC 19433, and *Lactobacillus acidophilus* ATCC 4356. The antimicrobial activity of the extracts was tested using the agar well diffusion method (Section 2.7) at concentrations of 100, 200, 300, and 400 µL/mL.

### 2.15. Statistical Analysis

Data were analyzed using IBM SPSS 25, Armonk, NY, USA. All phytochemical, antioxidant, antimicrobial, and color measurements were performed in triplicate (*n* = 3), and the results were expressed as mean ± standard deviation (SD). One-way ANOVA test was used to evaluate significant differences among treatments, followed by Duncan’s multiple range test to compare the means at a 95% confidence level (*p* < 0.05). An independent *t*-test was used to determine the significance of the antimicrobial activity of the gums. In addition, a correlation analysis was conducted using Spearman’s rank correlation coefficient and treatment mean values to examine the relationships between color parameters, antioxidant activity, and total phenolic content.

## 3. Results and Discussion

### 3.1. Extraction Yield

Ethanol extracted 23.33% phytochemicals from the SCGs (Table 2), consistent with the ethanol yields of 23.94% and 23.85% reported by Loyao et al. [22] and Linhares Sabino et al. [23], respectively. Ethanol extraction revealed a higher yield compared to other solvents such as water, acetone, isopropanol, and acetonitrile [11,24]. As a polar solvent, ethanol extracts high amounts of polar compounds along with low concentrations of oil [25]. Furthermore, ethanol interacts with the SCG matrix, breaking the hydrogen and hydrophobic bonds between phenolics and proteins, as well as cellulose, thereby enhancing yield. Nonetheless, Efthymiopoulos et al. [25] reported a lower ethanol yield (20.90%), which may be attributed to variations in coffee type, source, and extraction conditions, such as method, time, and temperature.

### 3.2. Total Phenolic and Flavonoid Contents of SCG Extract

The total phenolic content of the SCG extract was 999.38 µg/g, with flavonoids accounting for 14% of the total phenolics (135.44 µg/g) (Table 2). The extracted phenolics are comparable to 1.05 mg/g [16] and 91.83 mg/100 g [11] in SCGs extracts. However, the phenolic content was lower than 96.32 mg/g and 56.30 mg/g extracted from SCGs using 100% ethanol and ethanol: water 1:1 [24]. Furthermore, the flavonoid content in the present study was lower than that reported in previous studies: 62.50 mg/100 g [11], 1.55 mg/g, and 2.50 mg/g [24]. The variation between the results could be attributed to several reasons, including the coffee brewing process, the solvent used, and the extraction method. However, SCGs are considered rich in phenolic compounds.

### 3.3. DPPH Scavenging Activity of SCG Extract

The antioxidant activity of the SCG extract was determined, with an IC_50_ value of 107.28 μL/mL (Table 2). The high scavenging activity of the extract could be attributed to its high phenolic content. The antioxidant activity of the extract is more powerful than that reported in previous investigations. SCGs extracted using ethanol/water, ethanol/water/acetonitrile, and water showed an IC_50_ of 196.25 µL/mL [12], 0.16–0.80 mg/mL [24], and 1.04 mg/mL [11], respectively. Nonetheless, the finding is within the IC_50_ range, 10.10 to 301.80 µg/mL, obtained from different sources of SCGs extracted with ethanol [26]. The different results suggest that the extraction solvent and SCG source affect the antioxidant activity of the SCGs.

### 3.4. Cytotoxicity of SCG Extract

The concentration of SCG ethanol extract that inhibits the growth of WI-38 cells by 50% (CC_50_) was 386.40 μL/mL (Table 2). Previous investigations revealed the high selectivity of the SCG extracts. The extracts at low concentrations were cytotoxic towards carcinoma cells, while high concentrations were required to induce the cytotoxic effect on the normal cells [27,28]. The CC_50_ of the ethanol extract was close to the result obtained by the ethanol extract from caffeinated SCGs (CC_50_ > 400 μL/mL) on the primary pig liver culture cells (PLP2) [27]. However, the CC_50_ of the ethanol SCG extract, in the present study, was higher than that obtained using the SCG isopropanol extract on human healthy oral epithelial cells, with a CC_50_ of 133.70 μL/mL [28]. The finding reveals the safety of the extract, as a high concentration of the extract is required to cause significant damage to the WI-38 cells.

### 3.5. FT-IR of SCG Extract

**Figure 1:**
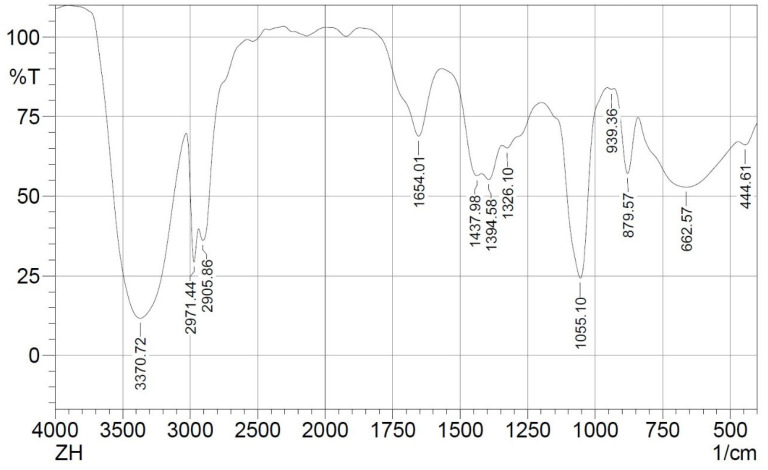
FTIR spectrum of SCG oily extract.

The FTIR spectrum of SCG extract reveals the presence of various functional groups (Figure 1). The spectrum showed a broad stretching vibration of a hydroxyl group (O-H) at 3370.72 cm^−1^ and an out-of-plane C-H bending of aromatic compounds at 879.57 cm^−1^ and 879.57 cm^−1^. Both stretching and bending vibrations are likely due to the presence of phenolic compounds within the extract.

Asymmetric C-H stretching vibrations were observed at 2971.44 and 2905.86 cm^−1^, denoting the presence of aliphatic hydrocarbons. Besides, bending vibrations at 1437.98 cm^−1^ and 1349.58 cm^−1^ suggest the presence of alkyl groups. The spectrum also shows a stretching vibration at 1654.01 cm^−1^ of the carbonyl group (C=O), possibly from carboxylic acids and esters. The peak at 1055.10 cm^−1^ could correspond to the C-O stretching of esters. These stretching vibrations suggest the presence of fatty acids and esters in the extract. Thus, the FTIR spectrum of the SCG extract indicates the presence of fatty acids, esters, and phenolic compounds in the ethanolic extract. The obtained vibrations in the present study are consistent with previous literature, particularly regarding the presence of fatty acids and esters vibrations [16]. Furthermore, the FTIR spectrum in this study also showed vibrations associated with phenolic compounds. This can be attributed to ethanol’s polarity, which facilitates the extraction of phenolics and flavonoids, along with some fatty acids [22].

### 3.6. Antimicrobial Effect of SCG Extract

**Figure 2:**
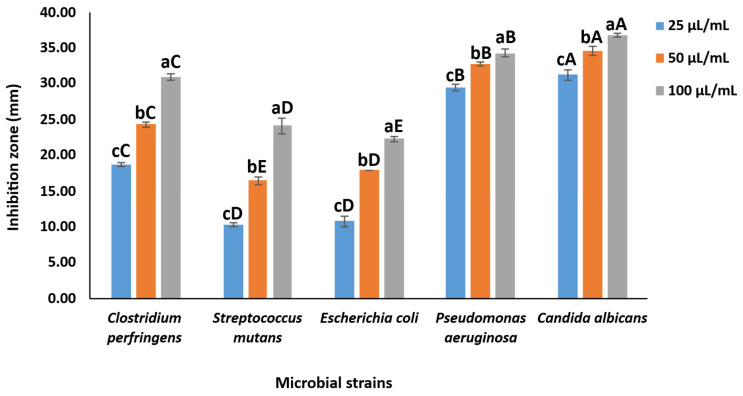
Inhibition zones (mm) of SCG extract against microbial strains. Different lowercase letters (a–c) indicate significant differences among SCG concentrations for the same microbial strain (*p* < 0.05). Different uppercase letters (A–E) reveal significant differences among microbial strains at the same SCG concentration (*p* < 0.05).

The solvent (control) did not produce any inhibition zone (0 mm) against the tested microorganisms. SCG extract reveals a potentiated and dose-dependent antimicrobial effect against the tested microbial strains (Figure 2). The extract (100 µL/mL) exhibited a potent inhibitory effect against *Candida albicans* and *Pseudomonas aeruginosa*, with inhibition zones of 36.83 ± 0.29 and 34.33 ± 0.58 mm, respectively. Moreover, 100 µL/mL of SCG extract inhibited the growth of *Clostridium perfringens*, *Streptococcus mutans*, and *Escherichia coli*, with inhibition zones of 31.00 ± 0.50, 24.14 ± 1.04, and 22.33 ± 0.29 mm, respectively.

Similarly, previous studies revealed that SCG extracts showed a promising antimicrobial effect against both G^+^ and G^−^ bacteria [11,24] and yeasts [11]. The high antimicrobial effect may be attributed to the high phenolic content of the extract (Table 1). The phenolic content can easily penetrate the cell membrane of G^+^ and accumulate in it, leading to membrane instability and cellular death [29]. Besides, the phenolics can interact with lipopolysaccharides and proteins in the outer membrane of the G^−^ bacteria, disrupting the membrane integrity and permeability [29]. Furthermore, the phenolics can also affect the membrane integrity and permeability of the yeasts, damaging the cell functions, and finally inhibiting their growth [30]. Thus, the antimicrobial effect of the extract may be attributed to the synergistic effect of the bioactive components in the natural extract, not to a specific phenolic compound [24]. The findings reveal the strong effect of the extract against the growth of Gram-positive (G^+^), Gram-negative (G^−^), and yeasts, suggesting the potential use of SCG extract as a potent antimicrobial agent in various food applications or drug-delivery systems.

### 3.7. Sensory Attributes of SCG-Enriched Chewing Gums

**Figure 3:**
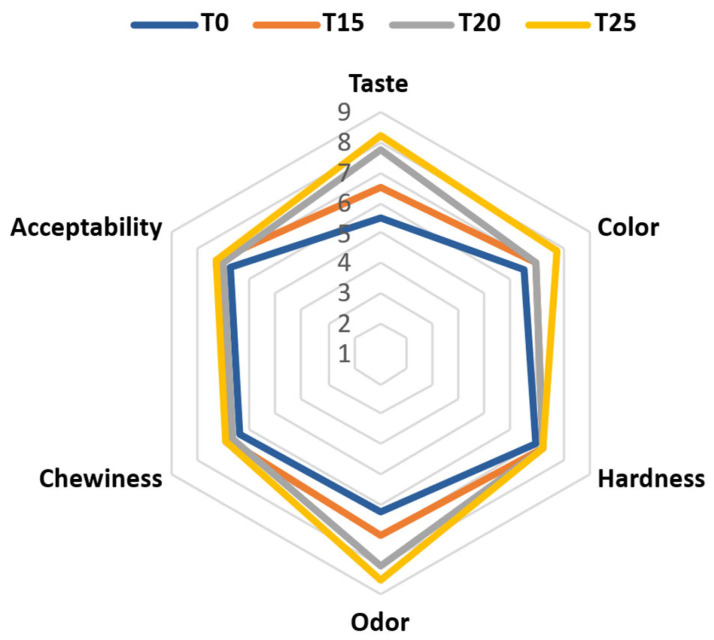
Sensory evaluation of gum enriched with SCG extract at 0% (T0), 15% (T15), 20% (T20), and 25% (T25).

SCG extract enhanced the taste, odor, and color of the SCG-enriched gums compared to the control (*p* < 0.05) (Figure 3). The panelists reported a coffee flavor in the enriched gums, which improved their taste and odor acceptability. The taste scored 8.25 ± 0.96 for T25 compared to 5.5 ± 0.58 for T0, and odor recorded 8.5 ± 1.00 (T25) versus 6.25 ± 0.50 (T0). The color of the extract positively influenced the acceptability of the gums’ color, with a score of 7.75 ± 1.50 for T25 compared to 6.5 ± 0.85 for T0. SCG extract improved the hardness, chewiness, and overall acceptability of the enriched gums, but not significantly, compared to the control. Lecithin and starch incorporated the SCGs into the gums and modulated the texture of the SCG-enriched gums, as in the control. Harness and chewiness scores ranged from 7.00 ± 0.85 (T0) to 7.25 ± 0.96 (T25) and 6.38 ± 0.48 (T0) to 6.88 ± 0.25 (T25), respectively. Besides, the overall acceptability of the SCG-enriched gums was slightly improved, reaching a score of 7.25 ± 0.96 in T25 compared to 6.75 ± 0.50 for T0. Thus, incorporating SCGs in gum production reveals a positive influence on the sensory attributes of the gums, which may be a successful functional food application and a possible drug-delivery system.

### 3.8. Color Parameters of SCG-Enriched Chewing Gums

**Figure 4:**
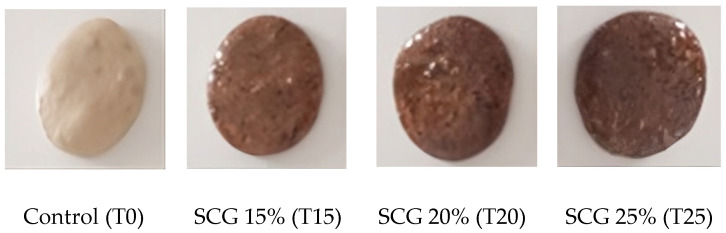
Chewing gums enriched with SCGs at different concentrations.

**Figure 5:**
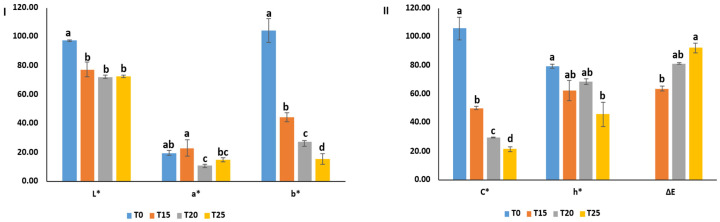
(**I**) L* (lightness), a* (Redness), and b* (Yellowness); (**II**) C* (chroma), h* (hue angle), and ∆E of gum enriched with SCG extract at 0% (T0), 15% (T15), 20% (T20), and 25% (T25). Different lowercase letters (a–d) indicate significant differences among SCG-enriched gum samples (*p* < 0.05).

Adding SCG extract significantly influenced the color parameters of the gums (Figure 4 and Figure 5). Lightness (L*) value of the SCG-enriched gums was decreased by nearly 21–26% compared to T0 (97.34 ± 0.67). However, increasing the extract concentration had no significant effect on L* values of the SCG-enriched gums, with values of 77.22 ± 4.96 (T15), 72.15 ± 1.24 (T20), and 72.51 ± 1.04 (T25) (Figure 5I). The darker color of the SCG-enriched gums compared to the control is attributed to the melanoidin pigments present in the SCG extract [31]. The similar L* values among treatments may result from the close ranges of SCG concentrations (15–25%). Besides, significant differences (*p* < 0.05) were observed in the a* and b* color parameters among the gum samples. The control gum (T0) showed the highest b* value (104.05 ± 8.026), while increasing the SCG concentration resulted in a progressive decrease in yellowness from 44.14 ± 3.06 in T15 to 15.32 ± 3.52 in T25. The a* value varied among the samples, being 19.46 ± 1.63 (T0), 22.97 ± 5.42 (T15), 10.78 ± 0.77 (T20), and 14.72 ± 1.23. Changes in a* values reflected alteration in the redness associated with the dark color of the SCG extract (Figure 5I). Consistently, SCGs addition has been reported to darken the color of different products, such as cookies, muffins, bread, pasta, and cake [1].

The chroma (C*), representing color saturation, and hue angle (h*), indicating color tone, were calculated to reflect the color purity and quality of the gum [32]. C* values of SCG-enriched gums decreased significantly by nearly 21–80% compared to the control, ranging from 50.01 ± 1.26 (T15) to 21.42 ± 1.81 (T25) versus 105.88 ± 7.89 (T0) (Figure 5II). Similarly, hue angle (h*) declined from 79.33 ± 1.59 in T0 to 62.51 ± 7.04 (T15), 68.55 ± 1.97 (T20), and 45.67 ± 8.57 (T25), reflecting a shift toward a darker hue. The color change (∆E) of the samples was observable because it exceeded the perceptible threshold of 3.5 [33]. ∆E values were 63.61 ± 1.74 (T15), 81.09 ± 0.54 (T20), and 92.27 ± 3.34 (T25), showing a slight increase with higher extract levels (Figure 5II). The findings reveal that incorporating SCG extract in gum production altered the gum color from light yellow in T0 to progressively darker brown shades in T15, T20, and T25 gums.

### 3.9. Phenolic Content of SCG-Enriched Chewing Gums

**Figure 6:**
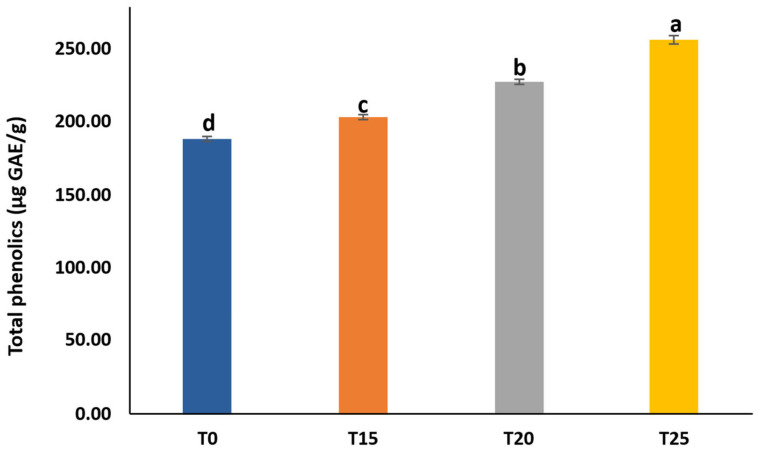
Total phenolic content of gum enriched with SCG extract at 0% (T0), 15% (T15), 20% (T20), and 25% (T25). Different lowercase letters (a–d) indicate significant differences among SCG-enriched gum samples (*p* < 0.05).

Total phenolics of the gums increased significantly with higher SCG extract concentrations (Figure 6). Control gum (T0) contained 189.02 ± 1.43 µg/g of total phenolics, which rose by approximately 8–36% in the enriched gums, reaching 203.53 ± 1.63 µg/g in T15 and 256.66 ± 2.93 µg/g in T25. This increase may be due to the high phenolic content of the SCG extract (Table 2). Generally, including phenolic-rich extracts in food applications enhances the bioactive potential of functional foods. Similarly, the addition of barberry [34] and eucalyptus leaf extracts [35] has been reported to increase the phenolic content of chewing gums compared to control formulations. Developing sustainable functional foods rich in phenolic compounds has gained increasing importance among health-conscious consumers. Thus, SCG-enriched gum, with its high phenolic content, may serve as an effective vehicle for delivering the bioactive components that support the management of various health conditions.

### 3.10. Antioxidant Activity of SCG-Enriched Chewing Gums

**Figure 7:**
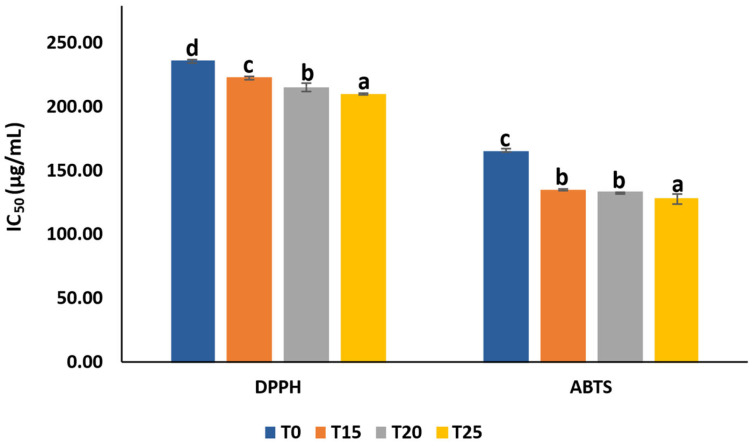
IC_50_ of DPPH and ABTS radicals of gum enriched with SCG extract at 0% (T0), 15% (T15), 20% (T20), and 25% (T25). Different lowercase letters (a–d) indicate significant differences among SCG-enriched gum samples (*p* < 0.05).

SCG extract markedly enhanced the antioxidant activity of the enriched chewing gums compared to the control (Figure 7). Adding SCGs to the gums reduced the amount of extract required to scavenge 50% of the free radicals. Control gum (T0) exhibited IC_50_ values of 236.92 ± 1.64 and 166.37 ± 1.07 µg/mL for DPPH and ABTS, respectively. These IC_50_ values decreased significantly with increasing SCG concentration in the gums by 6–11% for DPPH and 19–23% for ABTS, reaching 211.05 ± 0.65 µg/mL and 128.52 ± 4.05 µg/mL, respectively, in T25. The high antioxidant activity of the SCG-enriched gums can be attributed to the phenolic-rich SCG extract, which increased the phenolic content and antioxidant activity of the gums. Likewise, gums enriched with barberry [34] and eucalyptus leaf extracts [35] showed a potentiated antioxidant activity compared to control gums.

The findings revealed that SCG extract neutralized ABTS radicals more effectively than DPPH radicals, suggesting a broader spectrum of antioxidant mechanisms. While DPPH scavenging is based solely on electron transfer, ABTS scavenging relies on both electron and hydrogen atom transfer [36]. The potent antioxidant activity of the SCG-enriched gums may therefore contribute to reducing oxidative stress and mitigating related health disorders.

### 3.11. Correlation Between Color Parameters and Bioactive Properties

The correlation analysis further supports the close relationship between the color parameters of the gums and the bioactive compounds of the SCG extract (Table 3). Total phenolic content showed a strong negative correlation with b* value (ρ = −1.000), indicating that increasing SCG incorporation markedly reduced yellowness, which is consistent with the dark color of the extract. In parallel, a strong positive correlation was observed between total phenolic content and ∆E value (ρ = 1.000), reflecting the pronounced overall color difference between the control and SCG-enriched gums. Moderate negative correlations were also observed between total phenolic content and both L* value (ρ = −0.800) and a* value (ρ = −0.600), suggesting progressive darkening and changes in redness with increasing SCG levels. Regarding antioxidant activity, lower IC_50_ values correspond to higher effectiveness. Accordingly, the observed correlation patterns between color parameters and antioxidant activity, particularly between b* and ABTS/DPPH value (ρ = 1.000) and between ∆E and ABTS/DPPH value (ρ = −1.000), indicate that darker color attribute are associated with stronger antioxidant activity. These correlation results confirm that the compounds responsible for the dark color of the SCGs are closely associated with its functional properties.

### 3.12. Antibacterial Effect of SCG-Enriched Chewing Gums on Oral Bacteria

*Streptococcus mutans* is a key oral bacterium responsible for initiating dental caries. It metabolizes dietary sugars to produce exopolysaccharides that promote bacterial adhesion and biofilm (dental plaque) formation on tooth surfaces [20]. The bacterial community subsequently ferments sugars into acids, lowering the pH and leading to enamel demineralization and cavity formation [8]. *Lactobacillus acidophilus* further progresses the tooth decay by developing biofilms facilitated by the presence of *Streptococcus mutans*, which enhances its adhesion to the teeth [37]. Moreover, *Enterococcus faecalis* is associated with secondary and persistent endodontic infections, forming resistant biofilms within dentinal tubules and root canal walls [38]. Thus, developing antimicrobial delivery systems, such as chewing gums, may help inhibit these pathogenic bacteria and promote oral health.

**Figure 8:**
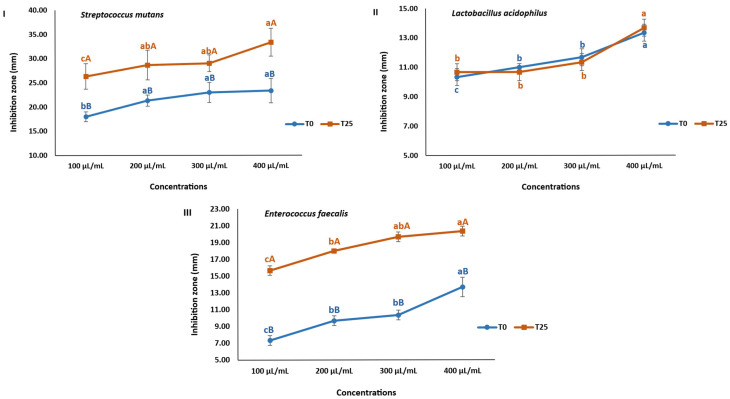
Inhibition zones (mm) of SCG-enriched chewing gums (T25) and control chewing gum (T0) against *Streptococcus mutans* (**I**), *Lactobacillus acidophilus* (**II**), and *Enterococcus faecalis* (**III**). Different lowercase letters (a–c) indicate significant differences among different concentrations for the same gum sample (*p* < 0.05); Different uppercase letters (A–B) reveal significant differences between gum samples at the same concentration (*p* < 0.05).

In the current study, the control gum (T0) revealed a notable antibacterial effect against oral bacteria, likely due to the use of frankincense resin as a gum base. Frankincense contains pentacyclic triterpenic acids, α and β-boswellic acids, and essential oils known to strongly inhibit the growth of various pathogens [9]. Additionally, sorbitol in the gum formulation may contribute to the antimicrobial effect [20]. Remarkably, incorporating SCGs significantly improved the antibacterial effect of the enriched gum (T25) compared to the control (Figure 8). SCG-enriched gum (T25) showed a potent antibacterial effect against *Streptococcus mutans* (Figure 8I) and *Enterococcus faecalis* (Figure 8III), along with a high inhibitory activity toward *Lactobacillus acidophilus* (Figure 8II).

The gum samples showed a strong inhibitory effect toward *Streptococcus mutans*, with inhibition zones (IZ) exceeding 15 mm [39]. T0 gum extract at concentrations of 100–400 µL/mL produced IZ ranging from 18.00 ± 1.00 to 23.33 ± 2.52 mm, which may be attributed to the natural gum base. Incorporation of SCGs in T25 significantly potentiated the antibacterial effect of the SCG-enriched gum compared to the control (*p* < 0.05), yielding IZ of 26.33 ± 2.65 to 33.33 ± 2.89 mm against *Streptococcus mutans* (Figure 8I).

For *Lactobacillus acidophilus*, SCG enrichment did not significantly alter the antimicrobial effect (Figure 8II). Both T0 and T25 demonstrated a modest inhibitory effect at 100 µL/mL (IZ = 10.33 ± 0.58 and 10.67 ± 0.58 mm, respectively), which increased at 400 µL/mL to 13.33 ± 0.58 and 13.67 ± 0.58 mm, revealing their high antimicrobial effect [39].

Regarding *Enterococcus faecalis*, T0 showed concentration-dependent inhibition, with IZ increasing from 7.33 ± 0.58 mm (at 100 µL/mL) to 13.67 ± 1.15 mm (at 400 µL/mL). SCG incorporation in T25 significantly improved antibacterial activity, achieving an IZ of more than 15 mm [39], ranging from 15.67 ± 0.58 mm (100 µL/mL) and 20.33 ± 0.58 mm (400 µL/mL) (Figure 8III).

Overall, SCG-enriched gum exhibited promising antimicrobial efficacy against oral bacteria, suggesting potential for reducing dental plaque formation and delaying caries onset. The findings are consistent with previous reports on the antibacterial activity of plant-based gums and extracts. For instance, propolis-enriched gum (4.75% in sugary gum; 5.06% in free-sugar gum) inhibited *Streptococcus mutans*, with an IZ near 7 mm [20], while propolis-based mouthwash also showed superior inhibitory activity against *Streptococcus mutans*, *Lactobacillus acidophilus,* and *Enterococcus faecalis*, compared to commercial mouthwash products [13]. In addition, apple juice was efficient in suppressing the growth of *Streptococcus mutans* and *Enterococcus faecalis*, with IZs of 12 mm and 9.40 mm, respectively [40]. The superior inhibition observed in the present study may result from synergistic effects of the gum components: frankincense resin, sorbitol, and the phenolic compounds in SCGs.

Thus, natural-based chewing gum enriched with SCG extract demonstrates significant potential as an antimicrobial oral care food product. However, further in vivo studies are necessary to substantiate these in vitro results.

## 4. Conclusions

Spent coffee grounds (SCGs) extract is rich in phenolic compounds, potentiating the antioxidant and antimicrobial effects of the extract. SCGs revealed a potent antimicrobial effect against various bacteria and fungi, which drew attention to using SCGs as an antibacterial agent. SCGs were successfully incorporated into the production of chewing gum up to 25%, using frankincense as a natural, biodegradable gum base. SCGs increased the phenolic content of the enriched gums and improved their antioxidant activity. Although the color change was noticeable, the sensory attributes of the developed gums were acceptable. Furthermore, gum enriched with 25% SCGs showed to be a promising product in monitoring the growth of harmful dental bacteria, *Streptococcus mutans*, *Lactobacillus acidophilus*, and *Enterococcus faecalis*. The SCG-enriched gum had a superior inhibitory effect on *Streptococcus mutans*, followed by *Enterococcus faecalis* and *Lactobacillus acidophilus*. Thus, the gum could support oral and dental health by inhibiting the growth of the bacteria. This effect could reduce plaque formation and dental caries development and progression. Further studies are required to validate the antibacterial effect of the SCG-enriched gums in in vivo models.

## Figures and Tables

**Table 1:** Formulations of SCG-enriched chewing gums (g/100).

	T0	T15	T20	T25
SCGs extract	0	15	20	25
Gum base	28	28	28	28
Sorbitol	57	42	37	33
Liquid sorbitol	15	13	12	10
Starch	0	1	2	3
Lecithin	0	1	1	1

**Table 2:** Physicochemical properties of SGC extract.

Yield (%)	23.33 ± 3.06
Total phenolics (µg/g)	999.38 ± 2.63
Total Flavonoids (µg/g)	135.44 ± 1.18
DPPH (IC_50_; µL/mL)	107.28 ± 1.90
CC_50_ (µL/mL)	386.40 ± 53.17

Data are expressed as mean ± SD.

**Table 3:** Spearman correlation coefficient between color parameters and bioactive properties of SCG-enriched gums.

Parameter	L*	a*	b*	∆E
TPC	−0.800	−0.600	−1.000 **	1.000 **
ABTS	0.800	0.600	1.000 **	−1.000 **
DPPH	0.800	0.600	1.000 **	−1.000 **

** Significant at *p* < 0.01 (Spearman’s rho); TPC (Total phenolic content).

## Data Availability

The original contributions presented in this study are included in the article. Further inquiries can be directed to the corresponding authors.
